# Carbapenem-Resistant *E. coli* Adherence to Magnetic Nanoparticles

**DOI:** 10.3390/nano14242010

**Published:** 2024-12-14

**Authors:** Oznur Caliskan-Aydogan, Chloe Zaborney Kline, Evangelyn C. Alocilja

**Affiliations:** 1 Department of Biosystems and Agricultural Engineering, Michigan State University, East Lansing, MI 48824, USA; oznurca@msu.edu (O.C.-A.); zaborne3@msu.edu (C.Z.K.); 2 Global Alliance for Rapid Diagnostics (GARD), Michigan State University, East Lansing, MI 48824, USA

**Keywords:** CRE, gMNP, MNP–cell interaction, rapid isolation, cell morphology, surface charge

## Abstract

Carbapenem-resistant *Enterobacterales* (CRE) is an emerging global concern. Specifically, carbapenemase-producing (CP) *E. coli* strains in CRE have recently been found in clinical, environmental, and food samples worldwide, causing many hospitalizations and deaths. Their rapid identification and characterization are paramount in control, management options, and treatment choices. Thus, this study aimed to characterize the cell surface properties of carbapenem-resistant (R) *E. coli* isolates and their interaction with glycan-coated magnetic nanoparticles (gMNPs) compared with carbapenem-susceptible (S) *E coli*. This study used two groups of bacteria: The first group included *E. coli* (R) isolates harboring carbapenemases and had no antibiotic exposure. Their initial gMNP–cell binding capacity, with cell surface characteristics, was assessed. In the second group, one of the *E. coli* (R) isolates and *E. coli* (S) had long-term serial antibiotic exposure, which we used to observe their cell surface characteristics and gMNP interactions. Initially, cell surface characteristics (cell morphology and cell surface charge) of the *E. coli* isolates were evaluated using confocal laser scanning microscope (LSCM) and a Zetasizer, respectively. The interaction of gMNPs with the *E. coli* isolates was assessed through LSCM and transmission electron microscope (TEM). Further, the gMNP–cell attachment was quantified as a concentration factor (CF) through the standard plating method. The results showed that the CF values of all *E. coli* (R) were significantly different from those of *E. coli* (S), which could be due to the differences in cell characteristics. The *E. coli* (R) isolates displayed heterogeneous cell shapes (rod and round cells) and lower negative zeta potential (cell surface charge) values compared to *E. coli* (S). Further, this research identified the differences in the cell surface characteristics of *E. coli* (S) under carbapenem exposure, compared to unexposed *E. coli* (S) that impact their attachment capacity. The gMNPs captured more *E. coli* (S) cells compared to carbapenem-exposed *E. coli* (S) and all *E. coli* (R) isolates. This study clearly found that differences in cell surface characteristics impact their interaction with magnetic nanoparticles. The gained insights aid in further understanding adhesion mechanisms to develop or improve bacterial isolation techniques and diagnostic and treatment methods for CRE.

## 1. Introduction

Antimicrobial resistance (AMR) is a global issue in emerging infectious diseases due to rapid demographic, environmental, and agricultural changes [1,2]. The overuse and misuse of antimicrobials and their release into the environment have created selective pressure on bacteria [3,4,5]. The sublevel antimicrobials in the environment slows their growth and promotes the selection of antimicrobial-resistant mutants, the transfer of their genes, and the evolution of de novo resistance [3,5,6,7,8]. Long-term low-level antimicrobial exposure modulates the microbial gene expression associated with cellular processes (carbohydrate metabolism, target modification, protein synthesis, etc.) [4]. This assists in the adaptation to new and unfavorable conditions and the emergence of new microbial phenotypes [4,6,8] for microbial survival at levels above the minimum inhibitory concentration (MIC) [3,6,7,9]. Microorganisms can acquire resistance via their rapid multiplication (vertical evolution) and horizontal gene transfer (HGT) among the same or different bacterial species or strains [4,5,10,11,12]. Therefore, the emergence and spread of AMR are alarming and pose a threat to the medical community and the public [2,4,13,14,15].

In the last decade, carbapenem-resistant *Enterobacterales* (CRE) have been listed as critical-priority AMR infections by the World Health Organization (WHO) and the Centers for Disease Control and Prevention (CDC) due to limited treatment options [13,16,17,18,19]. CRE mainly becomes resistant as a result of chromosomal mutations (non-carbapenemase-producing) and/or the production of carbapenemases (carbapenemase-producing). The emergence and spread of CRE are usually due to the rapid dissemination of genes encoding carbapenem-hydrolyzing enzymes (carbapenemase) through HGT, which accounts for 30% of CRE [2,20,21]. The most prevalent carbapenemases in CRE are listed as *Klebsiella pneumoniae* carbapenemase (KPC), Verona integron-encoded metallo-β lactamase (VIM), oxacillinase-48 (OXA-48), and New Delhi metallo-β-lactamase (NDM) [2,12,22], resulting in severe infections and colonization [11,17,21,23]. Several microbiological studies have found carbapenemase-producing (CP) CRE in biological and clinical samples [2,4,12,23,24,25,26,27]. Thus, the rapid identification and characterization of the causative bacteria are crucial in developing and implementing control and management options and treatments with the best antibiotic choices.

While various methods for identifying and characterizing antimicrobial-resistant bacteria (ARB) from pure culture and matrices have been developed [2,9,28,29], the literature hardly documents bacterial attachment properties or cell surface characteristics for bacterial isolation. Hence, this work aimed to assess the isolation of ARB, particularly CP-CRE isolates.

The existing methods for bacterial separation include physical methods (filtration and centrifuge, etc.) and chemical and biological methods (metal hydroxides, dielectrophoresis, magnetic nanoparticle (MNP)-based separation, etc.) [30,31,32]. Among these, MNP-based methods have commonly been utilized for the rapid and effective extraction of bacteria. MNPs have drawn attention due to their cost-effectiveness, stability, benign nature, and biocompatibility with detection assays [31,33,34,35]. One of the most significant advantages of MNPs is their superparamagnetic properties, assisting with rapidly dispersing MNPs in liquids; these can be magnetized and manipulated by an external magnetic field [31,33,36,37]. Another beneficial property is their high surface area/volume ratio, which results in a higher adsorption capacity, leading to potentially high binding affinity with the target. MNP–bacterial cell attachment relies on adsorption and chemical or biological interactions between specific surface substrates on a solid support and affinity ligands [30,31,38]. The affinity agents can be antibodies, bacteriophages, proteins, carbohydrates, and charged particles that interact through electrostatic interactions, hydrogen bonding, and Van der Waal’s forces [30,31]. These separation techniques can be nonspecific or specific to a target [31,38]. Therefore, the cell surface characteristics of bacteria and MNPs are important for efficient bacterial attachment.

In MNP-based separation, immunomagnetic separation (IMS) is commonly used for rapid selective separation. However, the lack of standardization, high experimental cost, and required storage conditions are primary limitations [30,31]. Recently, the use of glycan-coated MNPs (gMNPs) has offered several advantages: (1) a simple one-pot synthesis, allowing large-scale production abilities; (2) stability at room temperature with longer shelf-life, eliminating issues such as affordability and low-resource accessibility; (3) the glycan-based interaction of gMNPs allows its application in several bacterial types [28,31,39], making gMNPs lucrative alternatives. However, the application of gMNPs for ARB extractions has rarely been evaluated; differences in the attachment properties of carbapenem-resistant bacteria were observed in our previous work [28]. Thus, this study aimed to thoroughly investigate the cell surface characteristics of carbapenem-resistant *E. coli* isolates and carbapenem-induced (exposed) *E. coli*, along with their capability to adhere to gMNPs.

### Platform Novelty

Despite the increasing global burden of CRE infections, there is a considerable knowledge gap regarding bacterial extraction, including MNP–bacterial cell interactions. Therefore, this study aimed to expand the body of knowledge on the interaction of gMNPs with CRE, specifically carbapenem-resistant *E. coli* isolates, due to their prevalence worldwide [12,23,40,41,42,43,44].

This study evaluated two groups of *E. coli* isolates to further understand the gMNP–cell attachment mechanisms. The first group was composed of carbapenem-susceptible (S) *E. coli* and carbapenem-resistant (R) *E. coli* isolates harboring carbapenemases without carbapenem stress (exposure); their initial gMNP–cell binding capacity and cell surface characteristics were compared. The second group was composed of carbapenem-exposed and unexposed *E. coli* isolates (S and R1: KPC); their cell surface characteristics and gMNP–cell interaction were also compared. The cell surface characteristics in terms of cell morphology and surface charge were first examined. The gMNP–bacterial cell binding (adhesion) was then confirmed under a microscope, and their binding capacity was assessed using the concentration factor (CF) using the standard plating method. Further, the gMNP–cell interactions of *E. coli* (R) isolates in the absence and presence of carbapenem stress were compared with those of *E. coli* (S), along with their cell surface characteristics, and discussed.

## 2. Materials and Methods

### 2.1. Materials

Bacterial strains of KPC-producing carbapenem (ertapenem and imipenem)-resistant *E. coli* (BAA-2340) and *E. coli* C-3000 (15597) were obtained from the American Type Culture Collection (ATCC). Stock cultures of seven *E. coli* (R) isolates harboring carbapenemases were provided by the Michigan Department of Health and Human Services (MDHHS). Phosphate-buffered saline (PBS, pH 7.4), Tryptic Soy Agar (TSA), and Tryptic Soy Broth (TSB) were bought from Sigma Aldrich (St. Louis, MO, USA). Gram staining chemicals (Gram’s safranin solution, Gram iodine, crystal violet, and ethanol) were purchased from VWR International (Radnor, PA, USA). Further, transmission electron microscope (TEM) chemicals (glutaraldehyde, cacodylate buffer, and uranyl acetate stain) were provided by the Center for Advanced Microscopy (CAM), Michigan State University (MSU). TEM copper grids (formvar/carbon 200 mesh) were purchased from Electron Microscopy Systems (Hatfield, PA, USA).

### 2.2. Bacterial Groups in This Study (Study’s Approach)

The resistant profiles of *E. coli* (R) isolates from MDHHS were identified by growth-based AST and molecular (CDC laboratory-developed assay and CARBA-R Cepheid assay) methods at MDHHS. All *E. coli* (R) isolates and *E. coli* (S) were stored at −80 °C. The resistant and susceptible cultures were refreshed on TSA and then incubated at 37 °C for 24–48 h. For each experiment, fresh cultures were prepared in 9 mL of TSB with 4–6 h of incubation at 37 °C.

As previously mentioned, this study was conducted on two groups of bacteria in the presence and absence of the antibiotic, as listed in Table 1. The first group had several *E. coli* (R) isolates harboring carbapenemases and had no antibiotic exposure. Their initial MNP–cell binding capacity, with cell surface characteristics, was assessed. In the second group, one of the *E. coli* (R) isolates and *E. coli* (S) were chosen for long-term serial antibiotic exposure.

Carbapenem (imipenem) antibiotic was used for the second group of bacteria; imipenem is a broad-spectrum antibiotic against Gram-negative and Gram-positive bacteria and multi-resistant strains [45]. The breakpoint of imipenem for *Enterobacterales* is expressed as <1 µg/mL for susceptible, 2 µg/mL for intermediate, and ≥4 µg/mL for resistant cells in accordance with the Clinical & Laboratory Standards Institute (CLSI) [46]. Thus, the concentrations were defined as follows: low (0.25 and 0.5 µg/mL), intermediate (1 and 2 µg/mL), and high (4 and 8 µg/mL). First, carbapenem-exposed *E. coli* were created through serial long-term imipenem exposure on *E. coli* (S) to mimic low-level, medium-level, and high-level resistance in our previous work [47]. Briefly, *E. coli* (S) were separately plated in TSA with the various imipenem concentrations and incubated at 37 °C for a week. *E. coli* (S) were not able to survive at ≥2 µg/mL imipenem. The surviving cells at low concentrations (≤1 µg/mL of imipenem) were transferred onto TSA with the same antibiotic concentration for their serial cycles. With serial exposure at low concentrations, surviving cells were adapted to stress, and they reached their initial population in the first five cycles. The adapted cells at 1 µg/mL imipenem were then transferred onto TSA with 2 µg/mL imipenem, which were then able to grow and followed by their serial cycles. Similarly, the adapted cells at 2 µg/mL imipenem were transferred to TSA with 4 µg/mL imipenem at the end of the 5th cycle. Lastly, these adapted cells were transferred to TSA with 8 µg/mL imipenem to start their serial growth cycles. The serial cycles ended after 30 cycles. Also, *E. coli* (R1: KPC) were serially exposed to various antibiotic concentrations in the long term; they were able to grow at all concentrations [47]. The previous study showed the antibiotic tolerance and irreversible changes in physiological response, including the cell morphology of *E. coli* (S), with increasing imipenem concentrations for an extended period (during 30 serial growths) [47]. Thus, this study further used the exposed cells (after 30 cycles) to assess their cell surface charge and attachment properties.

### 2.3. gMNP Synthesis and Its Binding Capacity

The gMNPs (glycan-functionalized MNPs) were made of black iron oxide (Fe_3_O_4_) core and glycan (chitosan) shells in the Nano-Biosensor Laboratory, at MSU, following a previously documented procedure [48]. In brief, iron oxide nanoparticles were synthesized using ferric chloride hexahydrate (precursor) in a mixture of ethylene glycol (reducing agent) and sodium acetate (porogen). Deacetylated chitosan was used to surface-modify the iron oxide nanoparticles. The synthesized gMNPs (powder) were kept at room temperature; new synthesis was not necessary for each experimental day. The gMNPs (5 mg/mL) were suspended in sterile deionized (DI) water and sonicated for 30 min prior to analysis. Further, characterization of the synthesized gMNPs was conducted by assessing their structure and surface charge using a Zetasizer (Zen3600, Malvern, UK) and microscope, along with confirming their superparamagnetic properties (particle movement in an external magnetic field), following reported procedures [28].

**Scheme 1:**
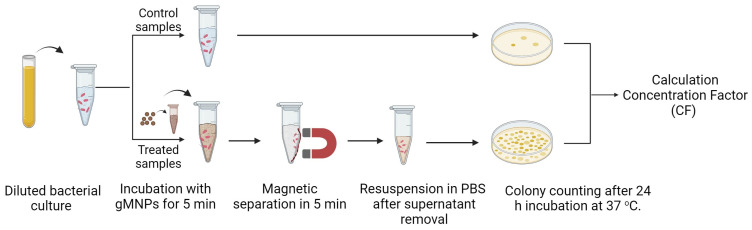
The procedure of gMNP–bacteria attachment capacity (created with BioRender.com, accessed on 10 November 2022).

The gMNPs–cell attachment capacity was then assessed through the standard plating method, as depicted in Scheme 1, which was adapted from an earlier study [28,39]. First, 5-h spiked fresh bacterial cultures were serially diluted in PBS at a concentration of 10^3^ CFU/mL and then plated for initial bacterial load as control. For the experimental group, 900 µL of the diluted sample (10^3^ CFU/mL) was mixed with 100 µL of gMNPs for 10–15 s and incubated for 5 min. After 5 min of magnetic separation, supernatant removal was followed by resuspension in PBS (100 µL). The suspended samples were finally plated on TSA and incubated at 37 °C for 24–48 h. For the carbapenem-exposed cells, TSAs included with the antibiotic concentrations were used. All experiments were conducted in triplicate.

The attachment capacity was defined as concentration factor (CF) by colony counting. The CF was separately calculated for each isolate based on control and gMNP-treated samples following the formula [28,39].

$$Concentration\;Factor=\frac{number\;of\;colonies\;in\;treated\;sample}{number\;of\;colonie\;in\;control\;sample}$$

### 2.4. Zeta Potential (Cell Surface Charge)

The zeta potential of the pure gMNPs and bacteria isolates was measured using a Zetasizer, following an earlier procedure [28]. First, 100 µL of gMNPs was suspended in 900 µL of sterile deionized water in a folded capillary cuvette, which was followed by measurement. For bacterial isolates, the consistency of the bacterial concentration of the freshly grown cultures was confirmed using optical density (OD 600: ~0.5) by the NanoDrop One C (Thermo Fisher Scientific, Madison, WI, USA). The bacterial isolates (~10^7^ CFU/mL) in TSB were centrifuged at 10,000 rpm for 3 min. The supernatant removal was followed by the resuspension of 1 mL of sterile DI water. The samples were then loaded into the cuvette and placed onto the Zetasizer. The experiments were performed in triplicate.

### 2.5. Visualization of Bacterial Cells and gMNP–Cell Interaction

Visualization of pure gMNPs and bacterial cells as well as confirmation of the gMNP–cell attachment were performed with a 3D laser scanning confocal microscope (LSCM) (VK-X1000 Series, Keyence, Osaka, Japan) in Dr. Alocilja’s Nano-Biosensors Laboratory at MSU, and a transmission electron microscope (TEM) (JEM-1400 Flash, JEOL, Nieuw-Vennep, Tokyo, Japan) at the CAM, MSU. First, the gMNP solution (5 µL) was directly dropped onto copper grid or microscope slides and washed with distilled water, followed by air-drying prior to imaging [28].

For visualization of pure bacterial cells using LSCM, bacterial cultures of the first group and second group were refreshed in TSA plates with and without the antibiotic for overnight incubation at 37 °C; 2–3 of the chosen colonies on the plates were smeared on the microscope slides, and the Gram-staining procedure was applied. LSCM also allowed to visualize the gMNP–cell interactions. For the imaging, the final sample of the gMNP–cell mixture was resuspended in 100 µL of sterile water after magnetic extraction and supernatant removal. A total of 10–20 µL of the final sample was smeared on a glass slide, followed by Gram staining. Optical images of pure bacterial cells and gMNP–cell interaction from different spots (10–15) on the slides were taken under 100× magnification with 1.5× zoom [47].

For TEM imaging, a loop of colonies of the overnight pure culture from the TSA plates and obtained gMNP–cell mixture was separately dissolved in the fixative solution (glutaraldehyde (2.5%) in cacodylate buffer (0.1 M) and then 5 μL of the sample was dropped onto the copper grid. After negative staining with 5 μL of uranyl acetate (0.5%) and air-drying, the grids were placed into the specimen holder of the microscope; images were observed in the range of 5000–25,000× magnification [28,47].

### 2.6. Statistical Analysis

Each experiment was conducted in triplicate, and data were presented with average and standard deviations in each bar graph. The differences in the measured values (zeta potential and concentration factor) of each group were statistically analyzed using the Kruskal–Wallis H test followed by post hoc Dunn’s test at a 95% confidence interval.

## 3. Results and Discussion

### 3.1. Visualization of Bacterial Cells

**Figure 1:**
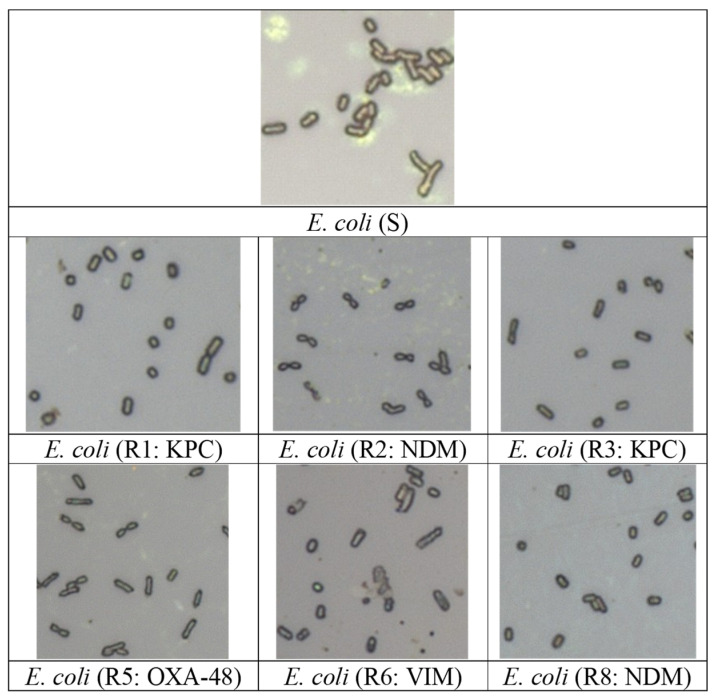
LSCM images of carbapenem-susceptible *E. coli* (S) and carbapenem-resistant *E. coli* (R) isolates harboring carbapenemases without carbapenem exposure (group 1).

The initial cell morphologies (cell shapes) of the *E. coli* (S) and all *E. coli* (R) isolates in the first group were monitored using LSCM, as illustrated in Figure 1. The rod (bacilli) shape of *E. coli* (S) was first confirmed. The cell structures or shapes of all *E. coli* (R) isolates were similar; these cells were heterogeneous, with nonperfect rod (bacilli) and round cells, unlike the susceptible cells, confirming the findings of earlier studies [28,47]. Additionally, a few studies showed that antibiotic-resistant cells’ biochemical components are different from those of susceptible cells based on distinct fingerprint patterns obtained by Raman spectroscopy [49,50]. Accordingly, the heterogeneity in the cell morphology of all *E. coli* (R) isolates might have resulted from differences in their cell wall components.

**Figure 2:**
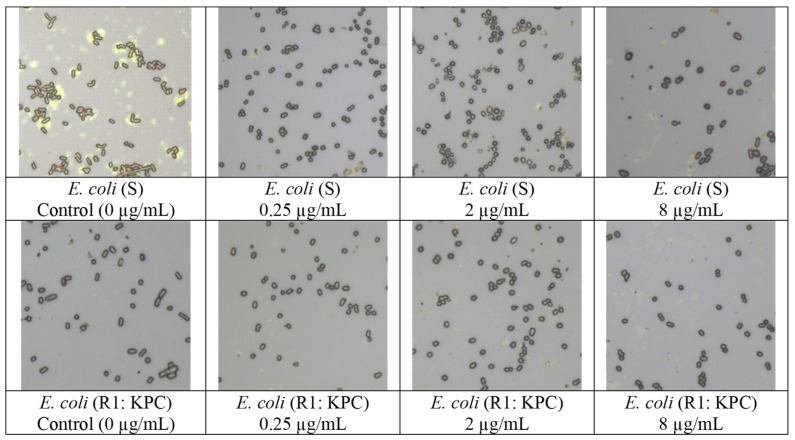
LSCM images of the carbapenem-exposed and unexposed *E. coli* (S) and *E. coli* (R1: KPC) cells at low, medium, and high- concentrations at the end of 30 serial growth cycles (group 2).

Further, the cell morphology of the control (unexposed) and the exposed *E. coli* cells of the second group at the low, medium, and high imipenem concentrations after 30 serial growth cycles were obtained and are depicted in Figure 2. As seen, the rod-shaped cells of *E. coli* (S) mostly became round cells after carbapenem exposure. In addition, *E. coli* (R1: KPC) under imipenem exposure was still heterogeneous (rod and round shapes); no major changes were observed. The ultrastructure of the carbapenem-exposed and unexposed *E. coli* cells was further confirmed using TEM in our previous work [47]; the images of the unexposed and exposed *E. coli* (S) clearly showed the round shape of the exposed cells with increasing imipenem concentrations. Overall, the exposed *E. coli* (S) cells, the exposed *E. coli* (R1: KPC), and unexposed *E. coli* (R) isolates similarly had both rod and round cells, confirming the findings of the previous study [47]. In addition, our previous work further characterized the size of exposed cells. The cell surface area-to-volume ratio (SA/V) of the exposed *E. coli* (S) was significantly different compared to the control, which was unexposed *E. coli* (S) [47]. The susceptible bacteria were possibly affected by antibiotic stress, resulting in their slower growth. Studies suggest that changes in cell components and morphology are interconnected with the bacterial growth rate [47,51,52,53,54,55,56,57]. Long-term serial carbapenem exposure with increasing concentrations gradually assisted bacterial survival under harsh conditions and modulated the cellular process [7,47].

Further, an earlier study evaluated the morphological changes of *Acinetobacter baumannii* exposed to meropenem and imipenem. The morphological changes were seen in resistant and susceptible cells; however, imipenem strongly affected the susceptible bacteria [58]. Elsewhere, the cell shape of Gram-negative bacilli exposed to carbapenems became spherical or ovoid cell forms in all strains of *K. pneumoniae*, *E. coli*, *P. aeruginosa*, *S. marcescens*, and *Proteus* spp. [59]. In another study, sensitive Gram-negative bacilli showed morphological changes with exposure to cephalothin, while naturally resistant bacteria were unaffected [60]. In addition, earlier studies indicated that the observed cell shapes remained stable with long-term repeated antibiotic exposure; the time scale of acquiring resistance was found to be 24 transfers [60], 20 days [61], and 1–15 days [3]. Accordingly, the similarity in the shape of the exposed *E. coli* (S) and all *E. coli* (R) isolates could be the reason for acquiring permanent resistance. As mentioned, our previous work further confirmed that the changes in cell surface structures after 30 serial cycles were irreversible, which was confirmed by regrowing them in imipenem-free media for 100 reverse growth cycles [47].

Furthermore, the observed morphological differences (heterogeneous shapes) could be due to the effect of carbapenems on the cell wall, disrupting cell wall synthesis. Carbapenem enters bacteria through porins and binds to penicillin-binding proteins (PBPs) to inactivate its enzymatic activity, weakening the glycan backbone in the cell wall [45,62,63]. This results in the loss of the peptidoglycan layer that provides rigid, shape-determining structure and membrane tension to cells [57,59,60,62]. Furthermore, antibiotics induce endolysin expression and explosive cell lysis, leading to vesicle formation in Gram-negative bacteria [64,65]. Also, antibiotic stress could induce some changes inside the cytoplasm [66]. Several studies stated that changes in the biosynthesis of cell wall components, membrane contents, and cytoplasmic components might result in disruptions in cell characteristics, such as cell morphology and surface charge [56,57,67,68,69]. In addition, differences in the cell wall composition of bacterial species can affect their cell adhesion properties (surface charges and specific binding locations) [31,33,52,70]. Thus, the observed morphological characteristics of carbapenem-exposed and carbapenem-resistant *E. coli* cells in this study are the result of distortion or differences in resistant cell components; the differences also might impact their motility and cell attachment to surfaces [28,47]. This study further assessed the cell surface characteristics (surface charge or zeta potential) of these isolates to obtain further insights into their cell surface properties and cell attachment capacity, specifically with nanoparticles.

### 3.2. Cell Surface Charge (Zeta Potential) of Bacterial Cells

**Figure 3:**
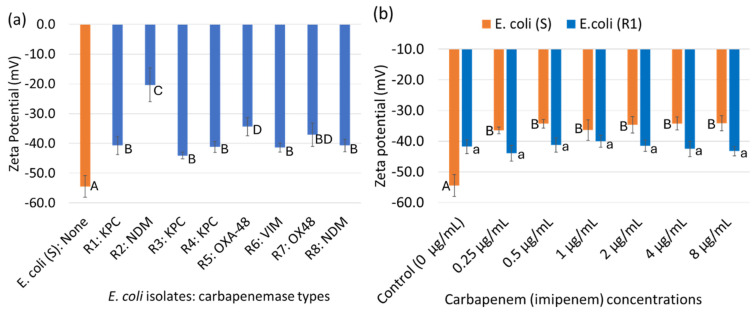
The mean zeta potential values of two groups of bacteria with standard deviations (N:27): (**a**) *E. coli* (S) and *E. coli* (R) isolates harboring carbapenemases, without carbapenem exposure (group 1) (capital letters represent statistical results) and (**b**) the long-term carbapenem-exposed and unexposed (control) *E. coli* (S) and *E. coli* (R1: KPC) at low, medium, and high concentrations (group 2) (letters represent statistical results: capital letters for comparison with *E. coli* (S) cells and lowercase letters for comparison with *E. coli* (R1: KPC) cells). Different letters above the bars denote significant difference (*p* < 0.05), and the same letter denotes no significant difference.

Zeta potential values were used as a proxy for cell surface charge. The average zeta potential values of all *E. coli* (R) isolates and *E. coli* (S) in the first group are illustrated in Figure 3a. The mean zeta potential value of *E. coli* (S) was around −54 mV, while those of all *E. coli* (R) isolates were between −20 mV and −43 mV. Herein, *E. coli* (R2: NDM) showed a significantly lower negative charge than all other *E. coli* (R) isolates. This could be the reason for the chemical nature of bacterial cells and the hydrophobic and hydrophilic groups on the cell walls, depending on bacterial type/serotype [28,56]. Overall, *E. coli* (R) cells had significantly lower negative zeta potential values (*p* < 0.05) compared to the reference bacteria (*E. coli* (S)).

The values of the unexposed and exposed *E. coli* (S) and *E. coli* (R1: KPC) in the second group are illustrated in Figure 3b. The exposed *E. coli* (S) at low, medium, and high levels of carbapenem were between -31 mV and −36 mV, while those of the unexposed *E. coli* (S) cells were around −54 mV. However, the unexposed *E. coli* (R1: KPC) and their exposed cells were in a similar range, approximately −43 mV to −40 mV. The zeta potential values of unexposed *E. coli* (S) and exposed *E. coli* (S) cells were significantly different (*p* < 0.05), while there was no significant difference between the unexposed and exposed *E. coli* (R1: KPC) (*p* > 0.05). The long-term imipenem-exposed *E. coli* (S) showed similar zeta potential values to those of all *E. coli* (R) samples regardless of carbapenem exposure. It could be concluded that carbapenem exposure can significantly influence the surface charge of susceptible bacteria but not that of resistant bacteria, similar to the morphological characteristics.

Cell surface charges are associated with multiple factors, including the outer membrane compounds on the cell wall, the tridimensional structure of the cell surface, environment pH, and ionic strength, among others [68,71]. Zeta potential is mainly linked with negatively charged functional groups accompanied by phospholipids, lipopolysaccharides, and proteins on Gram-negative cell surfaces, while it is accompanied by peptidoglycan, teichuronic acid, and teichoic acid on Gram-positive cell surfaces [71,72]. For instance, Soon and coworkers (2011) found that colistin-susceptible *Acinetobacter baumannii* cells exhibited a greater negative charge than colistin-resistant *Acinetobacter baumannii* cells [69], similar to our findings. They also indicated that the differences might be the result of lipopolysaccharide (LPS) loss in the outer-membrane proteins in resistant cells [69]. Further, another study stated that differences in lipid A structure on the cell walls reduce the negative charge of the cell, impacting their electrostatic repulsion [8]. Thus, the difference might be the reason for the loss of or the reduction in outer-membrane protein, porins, in carbapenem-resistant bacteria because carbapenem does not easily enter through the cell wall but diffuses through porins, as stated earlier [2]. Accordingly, the chemical nature of bacterial cells and potential modification in cells under any stress (environmental condition) lead to differences in growth rate, affecting cell wall composition, cell morphology, cationic and anion balance on the cell surface, electrical potential differences, and bacterial adhesion properties [56,67,68,71,72].

In addition, electrostatic surface charges are primed for the evolution of the bacterial adhesion process [68,71], utilized for interactions between bacterial cells and nanoparticles in several fields (biotechnology, chemistry, nanomedicine, pharmacology, etc.) [56,72], along with rapid bacterial extraction and detection in different matrices [28,29,31,33,56]. However, studies on the surface charges of ARB, including CRE, and their cell adhesion properties, are limited. Thus, this study examined the surface charge characteristics of a CRE (specifically *E. coli* isolates harboring carbapenemases), which were compared with reference (susceptible) bacteria, to provide further insights into their cell attachment properties, particularly with magnetic nanoparticles.

### 3.3. Characterization of the gMNPs and gMNP–Cell Binding Capacity (Concentration Factor)

**Figure 4:**
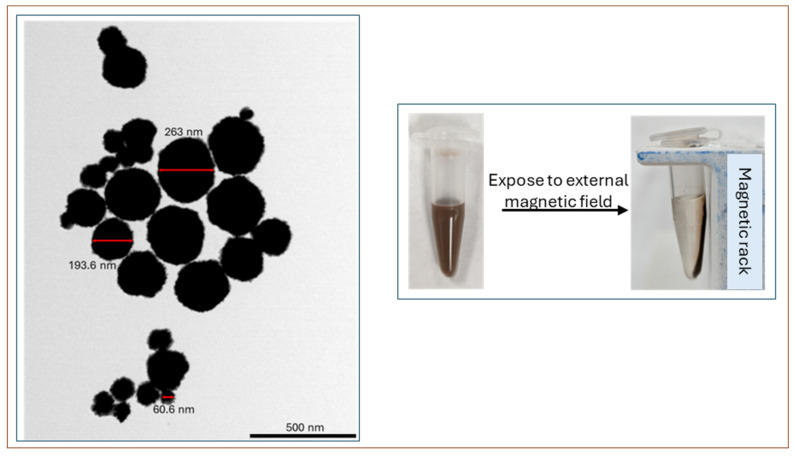
Characterization of synthesized gMNPs: TEM micrograph of gMNPs (**left**) and visualization of superparamagnetic properties of gMNPs under external magnet (**right**).

The synthesized gMNPs were first characterized in terms of morphology (shape, particle size (dimension), and surface charge) using the TEM and Zetasizer. As seen in the TEM micrograph in Figure 4, particle morphology is usually observed as roughly spherical with varying sizes (60–260 nm in diameter) and clumping of multiple particles. Further, the average particle size obtained with the Zetasizer was also approximately 268.5 ± 6.1 nm. In previous works, the size of the gMNPs according to TEM micrographs was similarly found to be 99 ± 58 nm [39], around 200 nm [70], and in the range of 40–300 nm in diameter [28], along with 286.5 ± 7.5 nm with the Zetasizer [28]. The size variation between the TEM and Zetasizer could be the effect of random clumping of multiple particles and the surface coating of iron oxide nanoparticles, as stated in earlier studies [28,39]. The particle size is significant, which provides a high surface area-to-volume ratio and superparamagnetic properties, improving bacterial capture [28,39]. For instance, a study showed that iron oxide nanoparticles 50–180 nm in diameter had the best superparamagnetic characteristics [73]. Further, Figure 4 confirms the superparamagnetic properties of gMNPs that were determined by exposing the solution to external magnetic area for a minute; the particles were all pulled out to the tube side, resulting in clear water, confirming previous findings [28,70]. Furthermore, the magnetization curves of the gMNPs were measured on a vibrating sample magnetometer by Dr. Alocilja’s group, confirming their superparamagnetic properties [74]. The magnetization saturation (Ms) properties of the gMNPs showed that the application of an external magnetic force rapidly saturated the MNP magnetic field, leading to gMNPs moving toward the magnetic force. Ms values can vary depending on the diameter of the ferric oxide core and functional group; Ms value of the gMNPs were found to be 47.06 emu/gm, showing enough magnetic strength [74]. Also, gMNPs do not aggregate without a magnetic field; thus, the superparamagnetic gMNPs are easily dispersed in an aqueous solution and move faster following microbial capture [70,74].

Further, the positive charge of gMNPs (+20 ± 6.1 mV) was confirmed, which creates electrostatic interactions with negatively charged bacterial cells for attachment [28]. The gMNPs have amino, hydroxyl and hydrophobic, and electron-rich regions, which interact with the positively charged bacterial surface, forming electrostatic force and non-covalent bond [70]. Previous studies showed that gMNPs were successful in capturing broad-spectrum bacteria such as *E. coli* O157, *S. aureus*, *L. monocytogenes*, *B. cereus*, *Salmonella* Enteritidis, *E. coli* C3000 [39,70,75,76,77]. Thus, this study further investigated the adherence of gMNPs to resistant bacteria.

**Figure 5:**
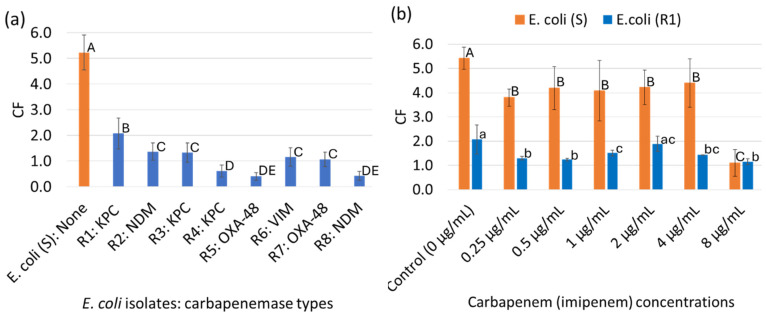
The gMNP–bacterial cell binding capacity, the average concentration factor (CF), of two groups of bacteria with standard deviations (N:18): (**a**) CF of *E. coli* (S) and *E. coli* (R) isolates harboring carbapenemases, without carbapenem exposure (group 1) (capital letters represent statistical results) and (**b**) the CF of the long-term carbapenem-exposed and unexposed (control) *E. coli* (S) and *E. coli* (R1:KPC) at low, medium, and high concentrations (group 2) (letters represent statistical results (capital letters for comparison of *E. coli* (S) cells and lowercase letters for comparison of *E. coli* (R1:KPC) cells). Different letters above the bars denote significant difference (*p* < 0.05), and the same letter denotes no significant difference.

The efficacy of gMNP binding with *E. coli* (S) and all *E. coli* (R) cells of the first group was initially investigated using the concentration factor (CF). All *E. coli* (R) isolates had a significantly lower CF (~0.4–2.1) than the CF of *E. coli* (S) (~5.3), as illustrated in Figure 5a. Overall, all *E. coli* (R) isolates had fewer bacterial cells compared to *E. coli* (S). However, it should be noted that the *E. coli* (R) isolates harboring the same carbapenemase had different CF values. It could be the result of differences in bacterial cell compounds that play the main role in their motility, cell surface charge, and receptor–ligand interaction; the bacterial attachment capacity can be variable based on bacterium type or serotype [28,31,39].

Further, the gMNPs were used to capture the unexposed and exposed *E. coli* cells; Figure 5b illustrates the CF values. The unexposed *E. coli* (S) had a higher CF value than the exposed *E. coli* (S), which were in the range of 4.6–1.1. The exposed *E. coli* cells at a high level (8 μg/mL) of carbapenem had the lowest CF, which could have been the effect of more disruption on their cell surface. Earlier studies showed that high-level antibiotic exposure produced more round cells and more cell damage [47,57,60]. The CF values of unexposed and exposed *E. coli* (R1: KPC) were closer, in the range of 1.6–1.2. Overall, gMNPs captured more *E. coli* (S) cells compared to the exposed *E. coli* (S) and all *E. coli* (R) isolates, which might have been related to the differences in the cell surface characteristics of the susceptible cells compared with the resistant cells and carbapenem-exposed cells.

In studies comparing nanoparticle–cell attachment capacity, for example, Gram-positive bacteria showed higher attachment to positively charged gold nanoparticles; the surface charge of Gram-positive bacteria was found to be more negative than that of Gram-negative bacteria [72]. In recent work, gMNPs were assessed on Gram-positive and Gram-negative bacteria: the CF (4.5) of *E. coli* O157 was lower than that of *S. aureus* (31.6) and *L. monocytogenes* (61.2). Herein, *E. coli* O157 had a lower negative surface charge (~−4 mV) than *S. aureus* and *L. monocytogenes* (~−42 mV and ~−35 mV, respectively) [39]. In another study, polyethyleneimine-modified magnetic microspheres (Fe_3_O_4_@PEI) were assessed for capturing bacteria using capture efficiency (CE); the average CE values of *S. aureus*, *P. aeruginosa*, and *A. baumannii*, and were found to be 97.87% 97.25%, and 80.57% respectively, which was related to their zeta potential values of −50.4 mV, −34.6 mV, and −23.9 mV, respectively [78]. In our study, *E. coli* (S) similarly had a higher CF, with a lower charge, and all *E. coli* (R) isolates had a lower CF with a higher charge. Our study further proved the established hypothesis that MNP–cell interactions are related to the zeta potential.

However, it should be noted that some of the *E. coli* (R5 and R7: OXA-48), (R1, R3, R4: KPC)) isolates had similar zeta potential values, but the CF values were significantly different. Further, *E. coli* (R2 and R7: NDM) isolates did not have similar CF or zeta potential values even though they both harbored the same carbapenemase. Herein, it should be remembered that MNP–bacterial cell attachment does not only rely on electrostatic interaction but is also based on receptor–ligand interactions [28,29,31,39]. Bacterial cell attachment to surfaces occurs via adhesins, such as polysaccharides in the cell membrane, cell wall, as well as capsule, and polypeptides (fimbrial (pili)) [79,80,81,82,83]. Bacterial types, regardless of susceptibility status, have unique components and attachment mechanisms; they mainly use fimbriae for attachment to host cells, but their length, type, and roles are different for each bacterial type, even for their serotypes [79,83,84]. Thus, the nature of bacterial cell surface components and the hydrophobic and hydrophilic groups on cell walls lead to differences in cell adhesion properties, including nanoparticle–bacterial cell attachment capacity [28,56]. Thus, differences in the CF within the *E. coli* (R) isolates could be additionally related to their serotype (unknown in this study) and more distortion in receptor–ligand interactions. Thus, this study used microscopes to confirm the gMNP–bacteria binding for further discussion of the adhesion mechanism.

### 3.4. Visualization of gMNP–Cell Interaction

**Figure 6:**
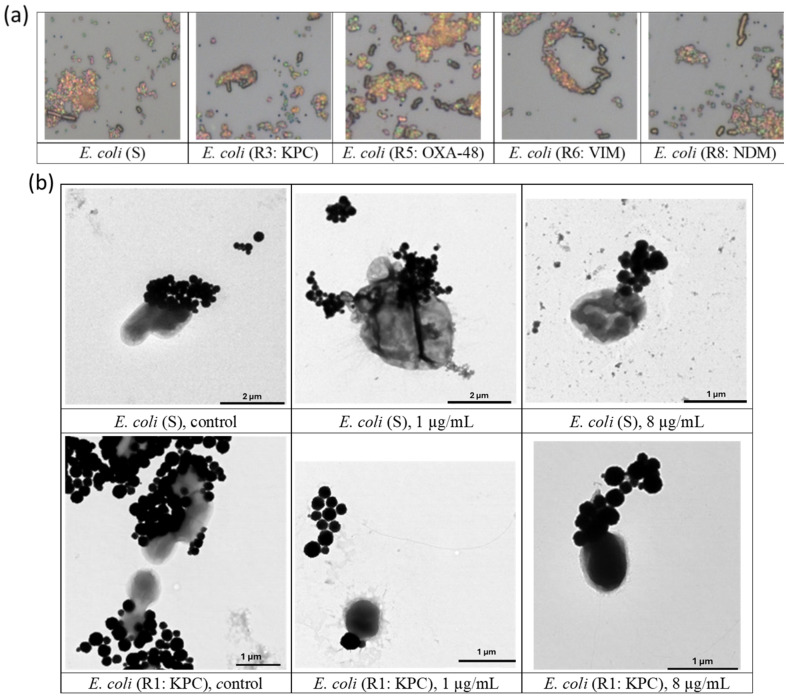
Microscopic images of gMNP–cell interaction of two groups of bacteria, which were obtained in the presence of gMNPs: (**a**) LSCM images showing the interaction of gMNPs with *E. coli* (S) and *E. coli* (R) isolates harboring carbapenemases, without carbapenem exposure (group 1) and (**b**) TEM images showing the interaction of gMNPs with long-term carbapenem-exposed and unexposed (control) *E. coli* (S) and *E. coli* (R1: KPC) cells at medium and high concentrations (group 2).

The gMNP–bacterial cell interactions were confirmed using LCSM. Figure 6a shows the successful binding of gMNPs to *E. coli* (S) and *E. coli* (R) cells in the first group. Clusters of susceptible and resistant cells attached to gMNPs were commonly seen, particularly in *E. coli* (R) isolates. Also, multiple gMNPs were attached to the individual cells.

Further, the gMNP–cell binding capacity of carbapenem-exposed and unexposed *E. coli* cells of the second group were visualized using TEM. As observed in the images (Figure 6b), the gMNPs similarly bound to carbapenem-exposed and unexposed bacterial surfaces. The presence of multiple gMNPs in a cluster or single cells might improve bacterial cell capture. The small size of gMNPs can aid in moving quicker than bigger particles, interacting with bacterial cells, and increasing the bacterial binding capacity [28,31,39].

The LSCM and TEM images further showed that the gMNPs mostly bound to some portions of the cell surface, not covering the entire surface of bacterial cells, which confirms that this is not only related to electrostatic charge difference. This behavior might be due to location-specific glycan–protein interaction, confirming our previous findings [28]. The MNP–bacterial cell interactions based on carbohydrates and cell surface proteins are established phenomena. For example, adhesion FimH, a two-domain protein, binds to terminal mannose residues [85]. Concanavalin A is another protein binding to sugar moieties in lipopolysaccharide [86]. Sugar moieties bind to C-type Salmo Salar Lectin (SSL) [87]. Site-specific attachment of bacterial cells was further illustrated in several studies using microscopic images [31,70,88].

**Scheme 2:**
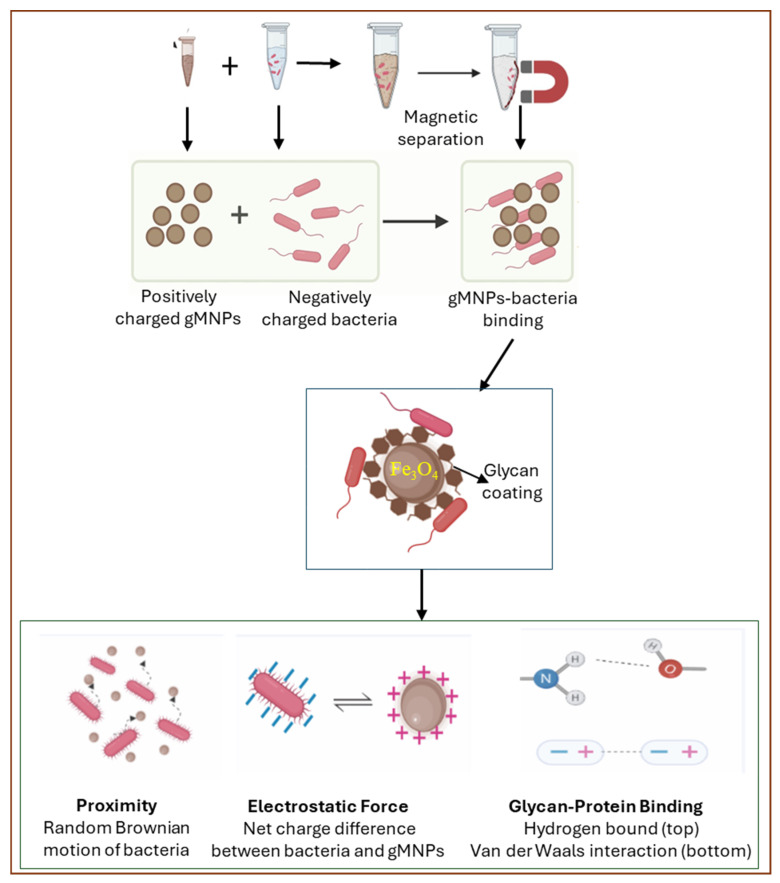
Demonstration of the hypothesis of the gMNP–bacteria attachment mechanism (created with BioRender.com, accessed in 2 November 2023), which was adapted from a study [28].

As previously studied, MNP–bacteria attachment relies on a combination of multiple forces: the random Brownian motion of bacteria (proximity), electrostatic interactions due to the charge differences between bacteria and MNPs, receptor–ligand (glycan–protein) interaction by van der Waals forces, and hydrogen bonds [28,39,89]. The attachment mechanism is further illustrated in Scheme 2. Chitosan (glycan) is a cationic biopolymer with a high percentage of amino groups, assisting with gMNP suspension in aqueous solution, which improves the proximity between the cell components and gMNPs. The polycationic structure of glycan easily binds to the anionic surface markers of bacteria. This study further found that these physicochemical interactions successfully allowed gMNP-resistant cell binding, similar to that of susceptible cells. However, it should be noted that the lower gMNP–cell capture in *E. coli* (R) isolates could have been due to the alterations in the morphological characteristics, cell surface charges, and the specific location of binding (glycan–protein interaction) [28]. As aforementioned, differences or alterations in lipopolysaccharide (LPS), peptidoglycan, and the outer membrane protein moieties of bacterial types can further impact their motility, surface charge, and receptor–ligand interactions [56,67,68,71,72]. For instance, the morphological characteristics of bacteria can impact their Brownian forces and cell attachment to surfaces [52]; the rod-shaped *E. coli* and coccus-shaped *L. monocytogenes* showed local binding in TEM images but had different attachment capacities [31,90]. Further, the interaction of gMNPs with *E. coli* 0157 and *E. coli* C3000 were separately evaluated; both individuals and clusters of both *E. coli* cells attached to gMNPs at mostly specific locations along with different attachment capacities (CF) and surface charges [28,39]. Overall, it should be noted that each bacterial type, even serotype, resistant bacteria, or bacteria in harsh environments may have different cell components (as chemical nature or defense mechanism), which could impact their adhesive properties such as motility, surface charge, and receptor–ligand interaction.

In addition, considering the effect of the localized heating and antimicrobial properties of MNPs on bacteria, the antimicrobial properties of chitosan-coated MNPs impacted a bacterial population due to long-term magnetic incubations (at least 8 h) [39,91]. A study also found that the localized heating of MNPs led to a significant decrease in the *S. aureus* population after antibody-coated magnetic nanocrystals were exposed to an *S. aureus* suspension under an alternating magnetic field for 1 h [92]. Thus, the experiment of short-term (~10 min: 5 min incubation +5 min magnetic field exposure) gMNP exposure at room temperature in this study would not have affected the bacterial population or their cell properties. Since the number of colonies from gMNP-treated bacteria is mostly higher than control (no gMNP treatment), the CF was higher than one. Also, images confirmed the similarities in the cell morphology before and after gMNP exposure. Further, earlier studies also confirmed that gMNPs were successful in concentrating bacterial cells in buffer solution and biological matrices; no significant effect was seen on the number of bacterial cells or their cell structure after magnetic extraction [28,39,74,75,76,77,93].

Further, the gMNPs can be used without further surface modification, do not require cold storage, and are chemically stable at room temperature, significantly reducing the cost [2]. The estimated material cost of gMNPs per assay was 0.5 USD [76,88], significantly lower than that of antibody-coated MNPs, which is estimated at 5–10 USD per assay [94]. In addition to its cost-effectiveness, bacterial extraction can be completed within 15 min [28,31]. Overall, the non-selective gMNPs allow the attachment of various bacterial species and resistant bacteria at once, which eliminates the need for specific functionalization for each bacterial type or species. The economical, efficient, and rapid nature of gMNPs and their storage conditions increase their applicability and accessibility for isolation techniques, especially in low-resource settings. However, future works are required to increase their applicability and accessibility.

### 3.5. Future Perspectives

The present study shows that gMNPs can rapidly capture ARB, including CRE (CP *E. coli*); the findings on gMNP–cell interactions and cell surface characteristics (cell morphology and cell surface charge) for susceptible and resistant *E. coli* cells further elaborated the bacterial characteristics and adhesive properties. While this study assessed several resistant *E. coli* isolates, they were compared with only one susceptible *E. coli*; thus, further studies with larger sample sizes are required. Each bacterial type/serotype, regardless of susceptibility status, has unique characteristics, adhesive properties, and relationships with hosts, which offer diverse genetic variability [95,96]. For instance, *E. coli* has various pathogen groups and serotypes (O- and H- antigens; >700 serotypes) based on virulent and antigen typing, respectively [97,98], each of which might affect their cell surface characteristics and interaction with surfaces. Thus, this study can be extended to include more susceptible *E. coli* isolates consisting of non-pathogenic and pathogenic types to elucidate if all susceptible *E. coli* cells behave similarly. In addition, other common carbapenem-resistant bacteria such as *Klebsiella*, *Enterobacter*, *Pseudomonas*, and *Acinetobacter* with their susceptible types and other causative ARB (vancomycin-resistant *Enterococcus*, ampicillin-resistant *Salmonella,* methicillin-resistant *Staphylococcus aureus*, colistin-resistant *E. coli*, etc.) should be assessed to further elucidate the behavior of resistant and susceptible cells.

Although this study showed that gMNP binding capacity to carbapenem-resistant and carbapenem-exposed *E. coli* (S) cells was lower compared to that of the control, *E. coli* (S), which could be related to cell wall components, further attention is needed to elucidate the interaction of gMNPs with bacteria and the surrounding environment. For instance, many bacteria can produce biofilm under any stress as their defense mechanism. Biofilm is typically a combination of polysaccharides, proteins, lipids, and DNA; bacterial cells in the biofilm matrix do not have Brownian movement [99,100]. Thus, future studies are needed to test the biofilm formation of bacteria in the absence and presence of antibiotic exposure, along with their interaction with gMNPs. Further, gMNPs can be tested in different matrices such as clinical, environmental, and food samples to understand the effect of matrix components and background microflora on the gMNP–bacteria adhesion mechanism. Moreover, although this magnetic separation using gMNPs is simple, rapid, and cost-effective, its efficiency can be further improved by coating with amine groups to increase their positive surface charge, which was shown to be capable of increasing the capture capacity of bacteria in food matrices [77].

While this study confirmed the differences in cell morphology and cell surface charge of the antibiotic-exposed and resistant cells, urgent attention is needed to explore their cell characteristics. Further research is needed to elucidate the mechanism of antibiotic exposure in bacterial cells. For instance, this study used only one carbapenem (imipenem); studies might be extended to use meropenem, which demonstrates enhanced activity against Gram-negative bacilli [101,102], and different kinds of antibiotics, such as cephalosporins and fluoroquinolones. The antibiotic-exposed bacteria may be genetically characterized to determine the emerging resistant genes.

The applicability of gMNPs may be extended to test in other fields. For instance, magnetic nanoparticles have been used in the medical field for diagnostics (nanoimaging and nanobiomarkers) and treatment (drug delivery system, nanosurgery, and gene nanotherapy) [92,103]. MNPs have been used in magnetic resonance imaging (MRI) for disease detection, utilizing their magnetic hyperthermia. Since magnetic nanoparticles are easily detectable by MRI, gMNPs can be further tested for optical imaging diagnosis [103]. In addition, the possible factors affecting gMNPs and bacterial populations should be considered; for example, temperature effect, exposure time, applied magnetic field intensity, or the effect of localized heating of MNPs can be further investigated. However, it should be noted that gMNPs are non-selective nanoparticles. For specific diagnosis or treatment, gMNPs can be additionally functionalized to have specific affinity for binding specific agents.

## 4. Conclusions

This study achieved our aim of investigating the cell surface characteristics of two groups of bacteria; carbapenem-resistant (R) *E. coli* isolates with susceptible (S) *E. coli* in the absence of carbapenem stress and long-term carbapenem-induced (exposed) *E. coli* (S) and one *E. coli* (R) with their unexposed cells, along with their adhesion capability with gMNPs. The applicability of gMNPs for the isolation of *E. coli* (R) compared with *E. coli* (S) was assessed. The cell morphology and surface charge were also evaluated to understand the gMNP–cell binding mechanisms. The gMNPs successfully bound to both *E. coli* (R) and *E. coli* (S) cells regardless of carbapenem exposure, which was confirmed with microscopic images and the standard plating method. However, the attachment capacity of all *E. coli* (R) isolates and the long-term carbapenem-exposed *E. coli* (R) and *E. coli* (S) isolates was significantly lower than that of the unexposed *E. coli* (S),control (reference). This could be the effect of the differences in cell surface characteristics; all *E. coli* (R) isolates without carbapenem exposure and the carbapenem-exposed *E. coli* cells displayed heterogeneous cell shapes (rod and round cells) and lower negative zeta potential (cell surface charge) values compared to the unexposed *E. coli* (S). This study clearly showed the differences in the cell surface characteristics between the resistant cells and exposed cells and the impact on their gMNP interactions. In future work, the use of the gMNPs can be tested on larger sample sizes, including more susceptible cells, CRE isolates, and other causative ARB isolates, and implemented in clinical and biological samples. These findings can stimulate future research on improving current extraction and diagnostic techniques.

## Figures and Tables

**Table 1:** Bacterial groups tested in this study.

(1) Carbapenem-Susceptible (S) *E. coli* vs. Carbapenem-Resistant (R) *E. coli*		(2) Carbapenem-Exposed *E. coli* vs. Unexposed *E. coli*	
Reference Isolate	Resistant isolates	No antibiotic treatment (Control, 0 μg/mL)	Antibiotic- treatment 0.25 and 0.5 μg/mL, 1 and 2 μg/mL, 4 and 8 μg/mL,
*E. coli* (S)	*E. coli* (R) R1: KPC (ATCC) * R2: NDM * R3: KPC R4: KPC R5: OXA-48 R6: VIM R7: OXA-48 R8: NDM	*E. coli* (S) *E. coli* (R1: KPC)	*E. coli* (S) *E. coli* (R1: KPC)

* The isolates have been tested in our previous work [28]. Here, they are only included to assess all other resistant *E. coli* isolates together. KPC: *Klebsiella pneumoniae* carbapenemase, VIM: Verona integron-encoded metallo-β lactamase, OXA-48: oxacillinase-48, and NDM: New Delhi metallo-β-lactamase.

## Data Availability

The data presented in this study are available upon request from the corresponding author.
